# FBXO15 plays a critical suppressive functional role in regulation of breast cancer progression

**DOI:** 10.1038/s41392-021-00605-4

**Published:** 2021-06-04

**Authors:** Yi Zhao, Nayeon Shim, Yan-Hong Cui, Jae-Hyeok Kang, Ki-Chun Yoo, Seungmo Kim, Joo Mi Yi, Min-Jung Kim, Jai Hoon Yoon, Su-Jae Lee

**Affiliations:** 1 Department of Life Science, Research Institute for Natural Sciences, Hanyang University, Seoul, South Korea; 2 Department of Medicine, Section of Dermatology, University of Chicago, Chicago, IL USA; 3 Department of Microbiology and Immunology, College of Medicine, Inje University, Busan, South Korea; 4 Laboratory of Radiation Exposure and Therapeutics, National Radiation Emergency Medical Center, Korea Institute of Radiological and Medical Sciences, Seoul, South Korea; 5 Department of Internal Medicine, College of Medicine, Hanyang University, Seoul, South Korea

**Keywords:** RNAi, Breast cancer, Breast cancer

**Dear Editor**,

Breast cancer is a leading type of cancer in women, the treatment of basal type, including triple negative breast cancer (TNBC), remains challenging due to its aggressive behaviors and lack of targeted therapeutic options^1^. F-box proteins (FBPs) can serve as either oncogenes or tumor suppressors depending on their substrates, and various studies have been conducted on their involvement in different types of cancer and play significant roles in cancer development and progression^2^. However, the related mechanisms and targets of F-box protein 15 (FBXO15) are still remain unclear in human breast cancers.

The TCGA and GSE21653 databases were used to screen a total of 69 FBPs to investigate their expression levels in luminal and basal type of breast cancer tissues. FBXO15 showed a more enrichment expression in luminal-type compared to basal-type of breast cancer patients (Supplementary Fig. 1a–c). Next, we assessed FBXO15 expression in different patient groups who were divided into PAM50 subtypes according to the RNA-seq or microarray data, and FBXO15 expression was well correlated with luminal-type compared to other subtypes or normal tissues (Fig. 1a). Another GSE41313 database and GSEA analysis using the GSE42568 database showed the consistent results (Supplementary Fig. 1d, e). Similarly, immunohistochemical staining revealed that FBXO15 protein expression was higher in normal tissues than in invasive ductal carcinoma (IDC) tissues (Fig. 1b); a higher FBXO15 expression level was observed in luminal-type compared to basal-type patients (Fig. 1c). Reanalysis of GSE21653 database and immunohistochemical staining also showed that decreased FBXO15 expression was observed in high-grade patient tissues (Supplementary Fig. 1f, g). Kaplan–Meier survival analysis released that an elevated level of FBXO15 expression correlated with a higher survival rate in patients, independent of their subtypes (Fig. 1d and Supplementary Fig. 1h).

**Fig. 1:**
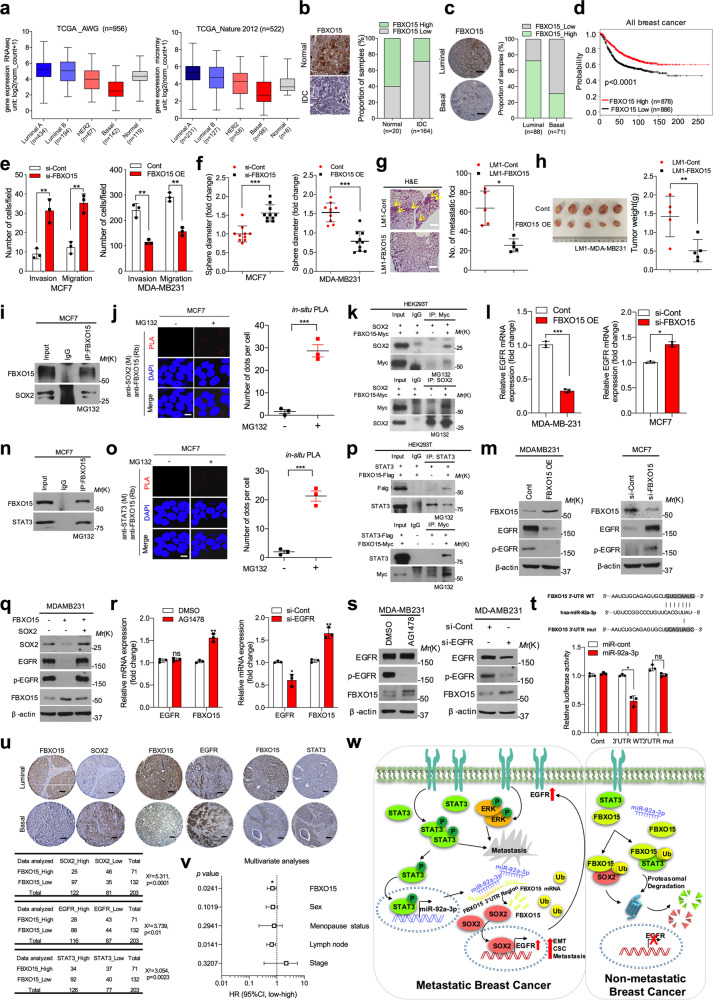
FBXO15 plays a critical suppressive functional role in regulation of breast cancer progression. **a** Log_2_ expression value of FBXO15 in breast cancer subtypes defined by PAM50 expression from RNA-seq (left) and microarray (right) data using the data from the TCGA_AWG and TCGA_Nature 2012 breast cancer database, respectively. **b** Representative IHC images of FBXO15 staining in normal and invasive ductal carcinoma (IDC) cancer tissues (left), and the graph shows the FBXO15-positive fraction of each group (right). The IHC test gives a score of 0 to 3 that measures the amount of FBXO15 protein staining in a breast cancer tissue. The score is 0 to 1 called low expression; the score is 2 to 3 called high expression. Image J software was used for analysis. **c** Images of IHC staining of FBXO15 expression in tissues from the luminal and basal types of breast cancer (left) and the proportion of FBXO15 expression is shown in the graph (right), scale bar = 100 μm. **d**. Kaplan–Meier survival analysis showed that high expression of FBXO15 corresponded with a better patient survival rate in all breast cancer patients. **e** Invasion and migration assays were performed to determine the number of cells after silencing FBXO15 in MCF7 cells or overexpressing FBXO15 in MDA-MB231 cells. **f** Sphere formation assay was performed using the same transfected cells, and the colony size were measured and shown in a graph. **g** Representative images of H&E staining of lung metastasis and the number of lung metastatic foci of each mouse with orthotopic xenografts. **h** Images of mouse tumors and the graph showed tumor weights from the control and FBXO15-overexpressed xenograft groups (*n* = 5 for each group). **i** Immunoprecipitation assay was performed, and the cell lysate was immunoprecipitated with an FBXO15 antibody or an immunoglobulin G (lgG) control to detect the endogenous protein interaction between FBXO15 and SOX2 in MCF7 cells. The cells were treated with MG132 (10 μM) for 6 h before harvest. **j** Representative confocal images and graph of cells with PLA-positive signal using MCF7 cells fixed with anti-SOX2 (M) and anti-FBXO15 (Rb). The graph showed the number of dots per cell which was counted using ImageJ software. Scale bar = 100 μm. **k** Co-immunoprecipitation assay indicating the interaction of FBXO15 and SOX2 using HEK293T cells. Cells were treated with MG132 (10 μM) for 6 h, and then, cell lysates were immunoprecipitated to pull down SOX2 and Myc-tagged protein. **l**, **m** QRT-PCR and western blotting analysis were performed to check EGFR or p-EGFR mRNA and protein expression levels after overexpressing or knock-down FBXO15 in MDA-MB231 and MCF7 cells, respectively. **n** Immunoprecipitation assay was performed, and the cell lysate was immunoprecipitated with an FBXO15 antibody or an immunoglobulin G (lgG) control to detect the endogenous protein interaction between FBXO15 and STAT3 in MCF7 cells. The cells were treated with MG132 (10 μM) for 6 h before harvest. **o** Representative confocal images and graph of cells with PLA-positive signal using MCF7 cells fixed with anti-STAT3 (M) and anti-FBXO15 (Rb). The graph showed the number of dots per cell which was counted using ImageJ software. Scale bar = 100 μm. **p** Co-immunoprecipitation assay indicating the interaction of FBXO15 and STAT3 using MG132 (10 μM) treated HEK293T cells. Cell lysates were immunoprecipitated to pull down STAT3 and Myc/Flag-tagged protein. **q** Rescue experiments were performed using western blotting analysis to assess EGFR and/or p-EGFR expression using FBXO15-overexpressing MDA-MB231 cells with or without SOX2 expression. **r**, **s** QRT-PCR and western blotting analysis of FBXO15 expression after silencing EGFR expression in MDA-MB231 cells using EGFR inhibitor, AG1478 (10 μM), or siRNA. **t** Graphic scheme of the miR-92a-3p binding to the 3’UTR region of FBXO15 (upper), and relative luciferase activity after wild-type (WT) FBXO15-3’UTR and mutant FBXO15-3’UTR co-transfection with miR-92a-3p in HEK293T cells (below). **u** Representative IHC staining images of FBXO15, SOX2, EGFR, and STAT3 expression in human breast cancer tissues. The tables showed the association between FBXO15 and SOX2, EGFR, STAT3 expression in breast cancer tissues. The association between FBXO15 and among SOX2, EGFR, and STAT3 expression in breast cancer tissues. The number of cases and the percentage of positive staining in the corresponding groups as well as the statistical significance based on Student’s *t* tests and Pearson’s correlations of expression are shown in the table. Scale bar = 100 μm. **v** Multivariate Cox regression analysis to regulate the significance of association between FBXO15 and disease-free survival (DFS) in the presence of other clinical variables. **w** Scheme of the EGFR/STAT3/miR-92a-3p/FBXO15/SOX2 axis mechanism in breast cancer. Scale bar = 100 μm, β-actin was used as a control for normalization of expression. *p < 0.05; **p < 0.001; ***p < 0.0001; ns not significant, determined by two-tailed Student’s *t* test (95% confidence interval).

To investigate the effects and mechanisms of FBXO15, we screened hallmarks of breast cancer using the GSE42568 database by GSEA analysis. We found that high expression of FBXO15 downregulated EMT and CSC pathways involved in cancer progression (Supplementary Fig. 2a). To confirm this finding, we knocked down FBXO15 in MCF7 luminal-type breast cancer cells. The cells showed a reduction in epithelial features and an increase in the mesenchymal phenotype, the invasion and migration potential, sphere size of colonies, and the percentage of CD44^+^/CD24^−^ cells. Furthermore, the signature genes expression of EMT and CSC were also increased both in FBXO15-silenced MCF7 and T47D cells. The inverse results were observed both in FBXO15-overexpressing MDA-MB231 and BT549 cells. (Fig. 1e, f and Supplementary Fig. 2b–g). Notably, overexpression of FBXO15 decreased cell growth and anchorage-independent growth (Supplementary Fig. 2h, i). In addition, xenograft models indicated that the tumor weight and lung metastatic foci number displayed a significant decrease in the FBXO15-overexpressing groups with inhibited the EMT/CSC signature genes, and proliferation marker Ki-67 expression (Fig. 1g, h and Supplementary Fig. 2j–n).

To investigate how FBXO15 inhibits malignant characteristics of breast cancer, we measured the main regulators expression of EMT (Snail, Slug, ZEB1, and Twist) and CSC (SOX2, OCT4, and NANOG). Finally, we found that SOX2 expression was increased at the protein level while there was no change at the mRNA expression level after silencing FBXO15 in MCF7 cells. Opposite results were shown in FBXO15-overexpressing MDA-MB231 cells and mouse xenografts (Supplementary Fig. 3a–i). To prove SOX2 would be a direct target of FBXO15, we performed CHX pulse chase assay and ubiquitination assay, found that decreased protein stability of SOX2 upon FBXO15 overexpression; ubiquitination of SOX2 was increased after co-expression with the FBXO15. The immunoprecipitation and in situ assay also showed an association between FBXO15 and SOX2 (Fig. 1i–k, and Supplementary Fig. 3j–l). Moreover, knockdown of SOX2 inhibited the EMT and CSC features of cells. The opposite results were observed after overexpression of SXO2, and these changes were reversed after co-overexpression of FBXO15 and SOX2 (Supplementary Fig. 3m–t).

Next, we performed GSEA analysis to investigate the signaling pathways dependent on FBXO15 expression and found that high expression of FBXO15 was negatively correlated with the EGFR-related signaling pathways, the TCGA database analysis also showed a negative correlation between FBXO15 and EGFR expression. Similarly, the total form of EGFR and p-EGFR expression were downregulated by FBXO15 (Fig. 1l and Supplementary Fig. 4a–c). Moreover, through screening the main downstream effectors of EGFR, we found that ERK and STAT3 signaling activation were negatively regulated by FBXO15. Interestingly, FBXO15 also downregulated the total form of STAT3 protein expression, rather than mRNA expression level (Supplementary Fig. 4d, e). The CHX assay, ubiquitination assay, co-immunoprecipitation, and in situ assays results showed that FBXO15 interacts with STAT3 to regulate its ubiquitination and degradation (Fig. 1n, o and Supplementary Fig. 4f–h). Previous study showed that SOX2 could regulate EGFR expression in neural precursor cells (NPCs)^3^, additionally, through reanalyzing the GSE21653 database and combination of our data, we found there was a positive correlation between SOX2 and EGFR. The ChIP assay further supported our hypothesis (Supplementary Fig. 4i–o). Collectively, FBXO15 controls EGFR expression and activation through regulation of SOX2 stabilization in breast cancer (Fig. 1q, and Supplementary Fig. 4p, q).

As reported, receptor tyrosine kinases (RTKs) play significant roles in the progression of metastatic breast cancer^4^. In order to investigate that whether FBXO15 expression is inhibited by EGFR in metastatic breast cancer. We blocked EGFR with inhibitor AG1478 or si-RNA, found the FBXO15 expression was increased and the reverse results were observed after overexpression of EGFR (Fig. 1r, s and Supplementary Fig. 5a). Additionally, knockdown of EGFR inhibited EMT- and CSC-related genes expression, while their expression was rescued when blocked EGFR and FBXO15 expression together. The opposite results were shown after overexpressing EGFR alone or together with FBXO15 (Supplementary Fig. 5b, c). With the inhibitors treatment of the main downstream effectors of EGFR, a significant increase of FBXO15 expression was observed after treatment with a STAT3 inhibitor compared to the other inhibitors treatment. Consistent results were shown in si-STAT3 transfected MDA-MB231 cells (Supplementary Fig. 5d, e).

To investigate how EGFR/STAT3 axis inhibited FBXO15 expression in basal-type breast cancer. DNA methylation analysis and screening of miRNAs which directly bind to the 3’UTR of FBXO15 were performed. Finally, we found that STAT3 inhibited FBXO15 expression not because of methylation (Supplementary Fig. 5f), instead, microRNA, miR-92a-3p, whose expression was high in basal-type breast cancer and associated with poor prognosis, and previous study also showed that miR-92a-3p can be induced by STAT3^5^, which consistent with our data. Furthermore, luciferase report assay confirmed that FBXO15 expression was negatively regulated by miR-92a-3p (Supplementary Fig. 5g–q, and Fig. 1t). Patients with *miR-92a-3p*^*high*^*/FBXO15*^*low*^ expression displayed a shorter survival time than those expressing *miR-92a-3p*^*low*^*/FBXO15*^*high*^ patients (Supplementary Fig. 5r).

IHC staining showed that FBXO15 expression was negatively correlated with SOX2, EGFR, and STAT3 expression (Fig. 1u). A multivariate analysis of survival based on Cox proportional-hazard model suggested that FBXO15 was an independent predictor of outcome in breast cancers (Fig. 1v).

In conclusion, our study elucidates that FBXO15 plays a key suppressive role in breast cancer through regulating STAT3 and SOX2 ubiquitination and degradation. Loss of FBXO15 expression leads to maintain the EMT and CSC phenotypes of breast cancer (Fig. 1w). Together, these findings reveal a novel and putative role of FBXO15 and inhibition of EFGR/STAT3/miR-92a-3p axis would provide a promising strategy for the treatment of metastatic breast cancer.

## Supplementary information

Supplementary Materials

## Data Availability

All data needed to evaluate the conclusions in the paper are present in the paper and/or the Supplementary Materials. Additional data related to this paper maybe requested from the corresponding authors.
