# Supplementary material for: FBXO15 plays a critical suppressive functional role in regulation of breast cancer progression
Source: Signal Transduct Target Ther. 2021 Jun 4;6:211. doi: 10.1038/s41392-021-00605-4 (PMC8175582; doi:10.1038/s41392-021-00605-4)
Supplement: Supplementary file 1 — Supplementary Materials [file 41392_2021_605_MOESM1_ESM.docx]

**Supplementary materials for**

**FBXO15 plays a critical suppressive functional role in regulation of breast cancer progression**

Yi Zhao^1^^†^, Nayeon Shim^1†^, Yan-Hong Cui^1,2^, Jae-Hyeok Kang^1^, Ki-Chun Yoo^1^, Seungmo Kim^1^，Joo Mi Yi^3^ , Min-Jung Kim^4^, Jai Hoon Yoon^5^, Su-Jae Lee^1,^*

*Correspondence to: Su-Jae Lee ([sj0420@hanyang.ac.kr](mailto:sj0420@hanyang.ac.kr))

**The PDF file incudes:**

Extended Discussion

Materials and Methods

Supplementary Text

Figures. S1 to S5

Reference

**Extended Discussion**

Breast cancer is the most frequently diagnosed cancer, leading cause of cancer death in women worldwide. It is worth noting that TNBCs are considered as the most aggressiveness and metastasis types than other types of breast cancers due to lack of targeted therapeutic possibilities ^1^. In this study, we demonstrated that FBXO15 is a putative tumor suppressor through regulation of SOX2 and STAT3 protein stabilization to suppress EMT and cancer stemness features of breast cancer. Moreover, we also found that the EGFR/STAT3/miR-92a-3p axis could downregulated FBXO15 expression to maintain the aggressive and metastatic phenotypes of TNBC. These findings reveal an unknown role and mechanisms of FBXO15 in regulation of breast cancer progression.

F-box proteins (FBPs) are the substrate subunit of Skp1-Cullin1-FBP (SCF) E3 ligases, which play significant roles in regulation of cellular function through degrading target proteins. FBXO15, as a member of FBPs, has been reported that it can interact with P-glycoprotein/ABCB1 to regulate its ubiquitination and degradation in anticancer drug resistance ^2^. However, the clinical function, mechanisms, and/or other targets of FBXO15 in human carcinomas are still unknown. In our research, we identified a novel target, SOX2, which can directly interact with FBXO15 in breast cancers. In line with GSEA analysis, FBXO15 expression regulates EMT and CSC-like features to induce metastatic phenotypes of breast cancer both in vivo and in vitro. Accumulating evidence has been reported that EMT-TFs make a promise to let cancer cells acquire stem cell properties for metastasis and dissemination ^3-5^; Additionally, CSC-TFs, such as, NANOG, and OCT4 also have been reported the roles in regulation of EMT and cancer stemness ^6,7^. Here, we found that EMT and CSC-TFs, such as Snail, Slug, and ZEB1 expression were increased at both mRNA and protein levels, while Twist, NANOG, and OCT4 expression have no change compared to si-control groups in FBXO15-silenced MCF7 cells. Interestingly, mRNA expression of SOX2 showed no obvious difference in si-control and si-FBXO15 groups. However, its protein expression level dramatically increased after silencing FBXO15 expression in MCF7 cells (Supplementary Fig. 3). Additionally, the results of cycloheximide (CHX) pulse chase assay, coimmunoprecipitation, and ubiquitination assays showed that FBXO15 directly interacted with SOX2 and accelerated its ubiquitination and degradation in breast cancer rather than NANOG and OCT4. In hence, we strongly suggested that FBXO15 directly interacts with SOX2, rather than other EMT-TFs or CSC-TFs in breast cancer. Although recent studies reported that SOX2 can be targeted by other E3 ligases, such as, WWP2 and TING-finger ubiquitin ligase complex CUL4A^DET1-COP1^ to maintain embryonic stem cell and neural progenitor cell differentiation ^8,9^. Nevertheless, in our study, we indicated a novel mechanism and function between FBXO15 and SOX2 in regulation of breast cancer malignancy.

Previous studies shown that EGFR, as a member of receptor tyrosine kinase family, expresses approximately 20-50-fold higher in tumors than normal tissues ^10,11^. Expression of EGFR shows a higher expression level in basal-type breast cancers or TNBCs than other type breast cancers ^12^. In line with recent work ^13^, as reported, EGFR plays a critical role in breast cancer progression, therefore, inhibition of EGFR expression and activation becomes a popular and efficient way in breast cancer treatment. In our research, the GSEA analysis showed that EGFR and its downstream signaling pathways displayed a negative correlation with FBXO15 expression in breast cancer. Furthermore, the western blotting analysis, ELISA assay, and qRT-PCR analysis data also demonstrated that FBXO15 could downregulated EGFR expression, activation and its downstream effectors activation, such as STAT3 and ERK signaling pathway. Importantly, the ubiquitination assay and co-IP assays data showed that FBXO15 also interacts with STAT3, a downstream effector of EGFR, to regulate its stabilization. Since SOX2, a transcription factor, regulates various downstream genes that are involved in cell proliferation, stem cell maintenance, and pluripotency ^14,15^. ChIP assay result released that SOX2 could directly bind to the promoter of EGFR to regulate its expression. When we blocked SOX2 expression in MDA-MB231 cells, the EGFR activity was also inhibited, performed by ELISA assay analysis. Therefore, our data suggested that FBXO15 could inhibit EGFR and its downstream effectors expression and activation through targeting SOX2 in breast cancer. Also, we indicated that FBXO15 expressed a higher level in luminal-type breast cancers than basal-type breast cancers and loss of FBXO15 expression induces a poor patients’ survival, independent subtype of breast cancers. Moreover, upstream regulation of FBXO15 screening showed that blocking of EGFR/STAT3 signaling pathway induces FBXO15 expression in basal-type breast cancer. As FBXO15 expresses a lower level in basal-type breast cancers not because of methylation, and previous study suggested that miRNAs function as post-transcriptional gene regulators through negatively regulate gene expression to mediate cancers invasion and migration ^16^, STAT3 activation could induce miR-92a-3p expression in human hepatocytes ^17^. We confirmed that STAT3 can regulate the expression of miR-92a-3p which consistents with the previous findings ^17^. Combination of these findings and previous studies, we firmly believe that EGFR/STAT3/miR-92a-3p axis downregulates FBXO15 expression in basal-type breast cancers or TNBCs. Additionally, tissue microarrays data also showed an inverse correlation among the FBXO15, SOX2, EGFR, and STAT3 proteins expression. Kaplan-Meier survival analysis indicated that high expression of FBXO15 and low miR-92a-3p expression in patients resulted in a better clinical outcome. Overall, in our research, we identify there is a negative feedback loop exits between FBXO15 and EGFR which associates with poor clinical outcomes in metastatic breast cancers. Certainly, there are also some insufficient sections in our study. First, our studies un-reveal the exactly binding domain of FBXO15 and/or SXO2, STAT3; second, whether the mechanisms and correlation between FBXO15 and EGFR/STAT3/miR-92a-3p axis also occurs in other human cancers are still unclear. Hence, further studies are required to determine FBXO15 roles in other human cancers.

In conclusion, our study elucidates that FBXO15 directly interacts with SOX2 and STAT3, ectopic FBXO15 expression induces their ubiquitination and degradation in breast cancer. In contrast, loss of FBXO15 expression promotes SOX2 and STAT3 protein stabilization, furthermore, SOX2 stabilization facilitates the EGFR/STAT3/miR-92a-3p axis activation, in reverse, inhibits the expression of FBXO15. Loss of FBXO15 function results tumor metastasis and poor prognosis outcomes of TNBC. Above all, FBXO15 plays a key suppressive functional role in regulation of TNBC progression through regulation of SOX2 and STAT3 ubiquitination and degradation. Targeting the EGFR/STAT3/miR-92a-3p axis to enhance FBXO15 expression provides an effective and possible strategy and mechanical system to relieve malignancies and improve the poor prognosis of patients with metastatic breast cancer.

**Materials and Methods**

**Cell culture**

MDA-MB-231, BT549, MCF7, and T47D breast cancer cell lines were purchased from the Korean Cell Line Bank (KCLB). The HEK293T cell line was from the American Type Culture Collection (ATCC), and LM1 cells were obtained from lung-metastasized MDA-MB-231 cells used for injection in mice. MDA-MB-231, 293T, and LM1 cells were cultured in Dulbecco’s modified Eagle’s medium, and BT549, MCF7, and T47D cells were grown in Roswell Park Memorial Institute medium from Gibco (Grand Island, NY, USA). All media were supplemented with 10% fetal bovine serum, penicillin (100 U/mL), and streptomycin (100 µg/mL). For sphere culture-conditioned medium, breast cancer cells were cultured in serum-free DMEM-F12 media (Invitrogen) as described previously ^15^.

**Chemical reagents and antibodies**

U0126 (#662005, a ERK1/2 inhibitor), SP600125 (#420119, a MAPK inhibitor), SB203580 (#559389, a P38 inhibitor), WP1066 (#573097, a STAT3 inhibitor), LY294002 (#154447-36-6, a PI3K inhibitor), AG1478 (#658552, an EGFR inhibitor), Cycloheximide (CHX, #66819), and MG132 (#474791, proteasome inhibitor) were purchased from Calbiochem (San, Pharmaceuticals, Inc., USA). Antibodies against STAT3 (sc-482), OCT4 (sc-9081), β-actin (sc-47778), AKT (sc-5298), SRC (sc-8056), p-ERK (sc-7383), JAK1 (sc-7228), and p-JAK1 (sc-101716) and normal mouse IgG (sc-2025) were purchased from Santa Cruz Biotechnology (Santa Cruz, USA). Vimentin (VIM, ab8978), fibronectin (FN, ab2413), 6x His-tag (ab16184), SOX2 (ab97959), CD44 (ab157107), and NANOG (ab21624) were obtained from Abcam (Cambridge, UK). Antibodies against Snail (3879S), Slug (7585S), EGFR (4267S), p-EGFR (3777S), p-AKT (4060), p-STAT3 (9131), p-SRC (2105), P38 (9212), p-P38 (9211S), and ERK (4695) were purchased from Cell Signaling Technology (Beverly, MA, USA). Antibodies against ZEB1 (HPA027524) and Flag (F1804-200UG) were obtained from Sigma (St. Louis, MO, USA). FBXO15 (NBP1-32860) antibody was purchased from Novus Biological (USA). Goat IgG antibody (HRP), rabbit IgG antibody (HRP), and mouse IgG antibody (HRP) were obtained from GeneTex (CA, USA).

**Transfection**

Vectors or siRNAs were introduced into cells using polyethylenimine (PEI) or Lipofectamine 2000 (Invitrogen), respectively, according to the manufacturer’s instructions. All siRNAs were purchased from Genolution Pharmaceuticals, Inc. (Seoul, Korea). All experiments were independently repeated three times with similar results.

**Western blot analysis**

Cell lysates were extracted with lysis buffer [40 mM Tris-HCl (pH 8.0), 120 mM NaCl, 0.1% Nonidet-P40] supplemented with protease inhibitors. Proteins were separated by SDS-PAGE and transferred to nitrocellulose membranes (Amersham, Arlington Heights, IL, USA). The membrane was blocked with 5% skim milk in Tris-buffered saline and incubated with primary antibodies at 4°C overnight. Western blots were developed with a peroxidase-conjugated secondary antibody, and proteins were visualized by enhanced chemiluminescence (ECL) procedures (Amersham, IL) according to the manufacturer’s protocol.

**Enzyme-linked immunosorbent assay (ELISA)**

For ELISAs, cellular protein was prepared according to the western blotting protocol. The concentration of phosphorylated EGFR was analyzed with human EGFR (pY1068) and total EGFR ELISA kits (Abcam, Cambridge, UK) according to the manufacturer’s protocol. All assays were repeated three times.

**RNA preparation and qRT-PCR**

Total RNA was prepared manually by using Tri-RNA reagent (Invitrogen, Carlsbad, CA, USA). A NanoDrop spectrophotometer (ND1000, NanoDrop Technologies, Wilmington, DE, USA) was used to assess RNA quality. All qRT-PCR analyses were performed using the SensiFASTTM SYBR No-ROX Kit from Bioline Reagents (UK) according to the manufacturer’s instructions, and reactions were carried out in a Rotor Gene Q system (Qiagen, Hilden, Germany). The results are expressed as the fold change calculated by the ΔΔCt method relative to the control sample. Beta-actin served as an internal normalization control. All primers were purchased from Macrogen (Seoul, Korea).

**Cycloheximide (CHX) pulse chase assay**

The CHX experiment was performed according to a previous report ^18^. Two days after transfection, the cells were treated with 100 µg/mL CHX solution (Sigma, USA) for the indicated time. Total protein lysate was collected at different intervals and subjected to immunoblotting.

**Co-immunoprecipitation**

SOX2 plasmids were cotransfected with Myc-tagged FBXO15 plasmids in HEK293T cells using Lipofectamine^TM^ 2000 Transfection Reagent. Two days after transfection, the cells were treated with 10 µM MG132 (EMD Millipore, USA) for 6 h. Then, HEK293T cell lysates were prepared by extracting proteins with cold lysis buffer [40 mM Tris-HCl (pH 8.0), 120 mM NaCl, 0.1% Nonidet-P40] supplemented with protease inhibitors. The cell lysates were precleared with Protein A-Agarose (Santa Cruz Biotechnology, USA), and the resulting supernatant fractions were incubated with appropriate primary antibodies overnight at 4°C. Immunoprecipitates were collected by incubation with Protein A-Agarose (sc-2001) for 2 h. Immunocomplexes were washed three times with 1X cold PBS and centrifuged at 6000 rpm for 2 min at 4°C. These immunoprecipitates were eluted from 1x SDS sample buffer and boiled. Then, the cells were analyzed by western blotting using the indicated antibodies.

**In situ proximity ligation assay (PLA)**

For the in situ PLAs, after 6 h of treatment with 10 μM MG132, cells cultured on a cover slip were fixed with 4% paraformaldehyde. The fixed cells were permeabilized with 0.1% Triton X-100 and 10% fetal bovine serum in PBS for 30 min and blocked with blocking buffer (5% goat serum and 2% BSA in 1X PBS) for 1 h. Then, the cells were incubated with anti-SOX2 or anti-STAT3 (1: 200) and anti-FBXO15 (1: 200) antibodies at 4°C overnight. In situ PLA was performed according to the manufacturer’s protocol using a Duolink Detection Kit (Sigma) with a pair of nucleotide-labeled secondary antibodies. Following ligation and amplification of the PLA probes, the signals were analyzed by confocal microscopy and quantified using ImageJ software (NIH, Bethesda, MD, USA).

**Ubiquitination assay**

A denaturing immunoprecipitation (IP) protocol was used to perform the ubiquitination assay. Specifically, HEK293T cells were transfected with His-Ub plasmid, FBXO15-Myc, SOX2, and STAT3 vectors. After 48 h, the cells were treated with 10 μM MG132 for 6 h to inhibit proteasomal degradation of SOX2. Subsequently, the cells were harvested by scraping and lysing using lysis buffer [denatured IP buffer: Tris–HCl (40 mM, pH 8.0), NaCl (120 mM), Nonidet-P40 (0.1%)]. IP buffer containing anti-SOX2 or anti-STAT3 was added to each sample to specifically pull down SOX2 or STAT3 protein, respectively.

**Migration and invasion assays**

For the migration assay, 2×10^4^ cells were seeded in the transwell with serum-starved medium, and the outside wells were filled with 10% FBS-containing medium. After incubation for 48 h at 37°C, the invaded cells on the outer surface were stained with a Diff-Quick kit (Sysmex, Japan) and photographed. Noninvaded cells on the inner surface were wiped off with a cotton swab. For the invasion assay, the assays were carried out as described for the migration assay except that each filter insert (Corning, USA) was coated with 10 mg/mL growth factor-reduced Matrigel (Corning, USA) and incubated at 37°C for at least 30 min before seeding the cells. Invasiveness was determined by counting cells in five microscopic fields per well, and the extent of invasion is expressed as an average number of cells per field. All experiments were repeated three times.

**Sphere formation assay**

For the sphere forming assay, the size of the transfected spheres was monitored after 7 days using Motic Images Plus 2.0 Software in three randomly chosen fields. Twenty to 30 spheres in each sample were measured from different independent fields, and the average size of the spheres was calculated.

**Flow cytometric analysis**

Flow cytometry was used to detect the CSC markers CD44 and CD24. A total of 1X10^6^ control cells and FBXO15-overexpressing or FBXO15 knockdown cells were harvested by trypsin digestion, washed and resuspended in 1X PBS. Then, the cells were incubated with R-phycoerythrin (PE)-conjugated anti-CD44 monoclonal antibody and FITC-conjugated anti-CD24 antibody (Miltenyi Biotec, Inc., Bergisch Gladbach, Germany) at 4°C for 30 min. All data were analyzed using CellQuest software (BD Biosciences) and repeated three times.

**Cell growth assay**

Cell growth was measured by directly counting the numbers of cells. Specifically, the cells were transfected with the control vector or the FBXO15 overexpression vector for 48 h. Then, 5×10^4^ cells were seeded into five 60 mm dishes. The total cell number of each group was counted at 24-h intervals.

**Soft agar colony formation assay**

Cell culture dishes (60 mm) were coated with 1.8% base agar (DifcoTM Agar Noble, BD, France) mixed with cell culture medium. A total of 1.5×10^5^ cells for each group were added to the mixture of 0.9% agar and culture medium on top of the base layer. The cells were incubated at 37°C for 7 to 10 days and analyzed as previously described ^19^.

**Luciferase reporter assay**

Luciferase reporter assays were performed using vectors encoding putative target sites in the 3' untranslated region (UTR). HEK293T cells were seeded into 60 mm dishes when after reaching approximately 50% confluency and were then cotransfected with reporter plasmid (1 μg), pRL-CMV-Renilla (Promega, Madison, WI) plasmid (1 μg) and miRNA using Plus reagent and Lipofectamine (Invitrogen) for 48 h. Luciferase activity was measured using a dual-luciferase reporter assay system (Promega) following the manufacturer’s instructions and normalized to Renilla luciferase activity.

**Chromatin immunoprecipitation (ChIP) assays**

Cells were crosslinked with 4% paraformaldehyde prior to performing the ChIP assay. ChIP assays were performed using an EZ-ChIP™ kit (EMD Millipore, Burlington, MA, USA) according to the manufacturer's instructions. IP was performed using an anti-SOX2 antibody or a rabbit isotype control IgG and RNA polymerase II positive antibody (Upstate Biotechnology, Lake Placid, NY, USA). GAPDH primer was used as a control. PCR was performed using primers specific to the EGFR gene promoter regions. For the normalization of ChIP-qPCR data, two common mthods are used, namely, the percentage of input and the fold enrichment. The percentage of input wsa calculated based on the equation 100 x 2^Ct (input)-Ct (Ip)^. The fold enrichment normalized to the IgG. SD was calculated from qPCRs performed in triplicate.

**Immunofluorescence**

For immunofluorescence staining, cells were fixed with 4% paraformaldehyde, permeabilized with 0.1% Triton in PBS and blocked for at least 30 min using blocking buffer (5% goat serum and 2% BSA in 1X PBS). The cells were then incubated with the appropriate primary antibody at 4°C overnight. The next day, after the cells were washed with blocking buffer (5% goat serum and 2% BSA in 1X PBS) 3 times, Alexa Fluor 488-conjugated anti-rabbit or anti-mouse and Alexa Fluor 546-conjugated anti-rabbit or anti-mouse (Molecular Probes, Eugene, OR, USA) secondary antibodies were used to detect the proteins, and 4′,6-diamidino-2-phenylindole (DAPI; Sigma, St. Louis, MO, USA) was used to stain cell nuclei. Cells were examined with an IX71 fluorescence microscope (Olympus, Tokyo, Japan).

**Immunochemical staining analysis**

Mouse tissues and patient tissues were fixed in formalin to generate paraffin sections. Paraffin-embedded tissue sections were deparaffinized in xylene and 100%, 95%, and 70% ethanol, followed by phosphate buffered saline (PBS). Epitopes were unmasked with 20 mg/mL proteinase K in PBS with 0.1% Triton X-100. The sections were stained with H&E or immunostained overnight at 4°C with primary antibodies. After PBS washes, biotinylated goat anti-rabbit IgG or anti-mouse IgG antibody was applied to the sections for 30 min. After a PBS wash, ABC reagent (Vector Laboratories, USA) was applied to the sections for 30 min. The color reaction was performed with 3,3'-diaminobenzidine (Vector Laboratories). After counterstaining with hematoxylin and clearing with a graded ethanol series and xylene, the sections were mounted with Canada balsam. Images were captured with a DP71 digital imaging system on an IX71 microscope (Olympus, Korea).

**DNA methylation analysis**

For methylation analysis, a standard phenol-chloroform method was used for genomic DNA extraction. Modification of 1 μg of DNA by bisulfite with the EZ DNA Methylation Kit™ (Zymo Research, Orange, CA, USA) guarantees a >99% conversion rate (of nonmethylated C nucleotides to U; protection of methylated cytosine residues). Methylation-specific PCR (MSP) primer pairs, which were located close to the putative transcription start site in the 5′ CpG islands, were used to analyze the methylation of gene promoters. A total of 2 μL of bisulfite-treated DNA was used as a template, and JumpStart REDTaq DNA Polymerase (Sigma-Aldrich Co.) was used for amplification as previously described ^20^. MSP primer sequences were as follows: *FBXO15* Unmethylation Forward 5′-GTAAAGTTATTTGGGAAATTTTTGT-3′ and Reverse 5′-AAAAATATAATCTCTTTTACACACC-3′; Methylation Forward 5′-GTAAAGTTATTTGGGAAATTTTCGT-3′ and Reverse 5′-AAAAAATATAATCTCTTTTACGCGC-3′. The amplified products were stained with ethidium bromide, resolved by 1% agarose gel electrophoresis, and photographed under UV illumination.

**Animal experiments**

All animal experiments were performed according to the guidelines of the Institutional Animal Care and Use Committee of Academia Sinica. NSG mice were obtained from Orient Bio (Seoul, Korea). A total of 40 µL (1×10^6^ cells) of LM1 control cells or FBXO15-overexpressing LM1 (lung-metastasized MDA-MB-231) cells was injected into the fat pad of 8- to 10-week-old female NOD/SCID mice (n=5 each group). After injection, the weight and tumor size of the mice were measured using a digital caliper. Tumor volumes were determined by measuring the length (l) and width (w), and the following formula was used for calculation: (shortest diameter^2^ x longest diameter/2). The mice were sacrificed 4–6 weeks after injection, and tumors and lungs were collected for biochemical studies.

**Human tissue microarray**

Human breast cancer tissue microarray (BR20814, BR2085c, BR2085d, Rockville, MD, USA) samples were purchased from US Biomax. These samples were reviewed by a pathologist to confirm the diagnosis of breast carcinoma, histological grade, and tumor purity. Images were captured using a DP71 digital imaging system on an IX71 microscope (Olympus, Tokyo, Japan).

**Gene set enrichment analysis (GSEA) dataset and Kaplan-Meier analysis**

GSEA was performed on diverse gene signatures by comparing gene sets from either the Molecular Signature Database (MSigDB) database or published gene signatures. Previously published microarray data under accession codes GSE41313, GSE21653, GSE42568, GSE352173, and GSE19783 were reanalyzed. The survival data were analyzed using the KM plot program (http://kmplot.com/analysis/) as previously described ^21^.

**Statistical analysis**

All experiments were performed at least three times, and the data are presented as the mean ± standard deviation (S.D.). The significance of differences was assessed by an unpaired two-tailed parametric Student’s t-test and one-way analysis of variance (ANOVA) using Prism 8.0 software (GraphPad, San Diego, CA, USA). Variances were confirmed to be similar between groups that were statistically compared, and p-values < 0.05 were considered significant.


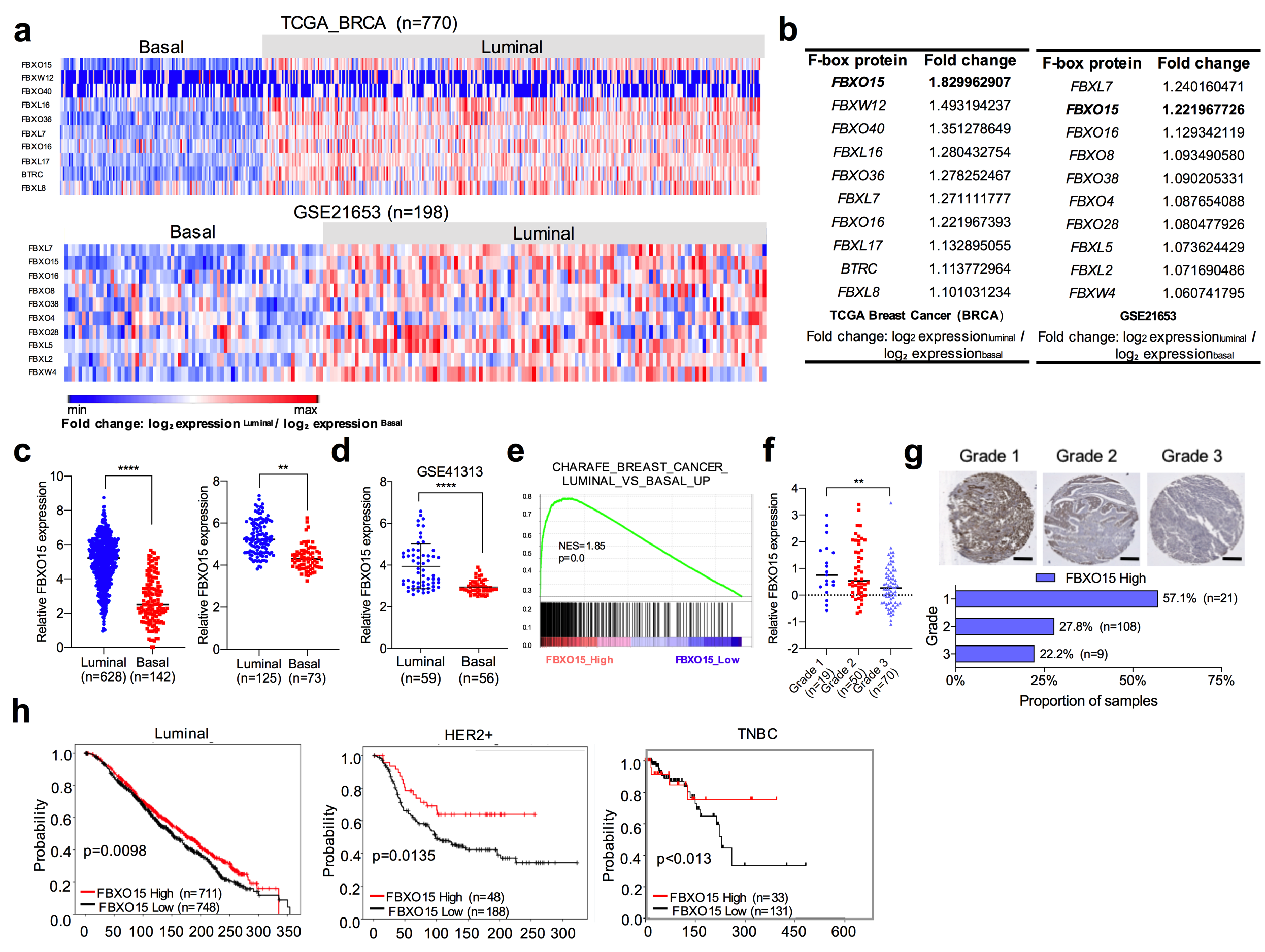


**Figure S1.** **FBXO15 displayed a low expression level in metastatic breast cancers.** **a.** Heatmap showing the F-box protein expression levels in patients with the luminal and basal types of breast cancer using data from the TCGA and GSE21653 breast cancer databases. Red and blue indicate upregulation and downregulation, respectively. **b.** The table showed the ranked list of top 10 F-box proteins expressed high level in luminal-type of breast cancer by fold change in descending order. using the data from TCGA and GSE21653 breast cancer database, respectively. The fold change calculated by log_2_ expression of luminal divided by log_2_ expression of basal type breast cancer. **c.** Graph showing FBXO15 expression in luminal and basal type of breast cancers using the data from TCGA (left) and GSE21653 (right) breast cancer databases, respectively. **d.** FBXO15 expression in cell lines from the luminal and basal subtypes of breast cancer using the GSE (GSE41313) database. **e.** GSEA analysis revealed that FBXO15 expression was positively correlated with luminal type breast cancer (GSE42568). NES, normalized enrichment score. **f.** A negative correlation between FBXO15 expression and breast cancer grade levels was observed using the data from GSE21653 database. **g.** Tissue microarray analysis of FBXO15 expression in different grades of breast cancer (upper), and the proportion is shown in the graph (below). The IHC test gives a score of 0 to 3 that measures the amount of FBXO15 protein staining in a breast cancer tissue. The score is 0 to 1 called low expression; the score is 2 to 3 called high expression. Image J software was used for analysis. **h.** Kaplan-Meier survival analysis showed that high expression of FBXO15 corresponded with a better patient survival rate in luminal-type, HER2^+^-type, and TNBC patients. Scale bar = 100 μm. The Metabric and GSE21653 public databases were used for reanalysis. **p < 0.001, and ****p < 0.0001.


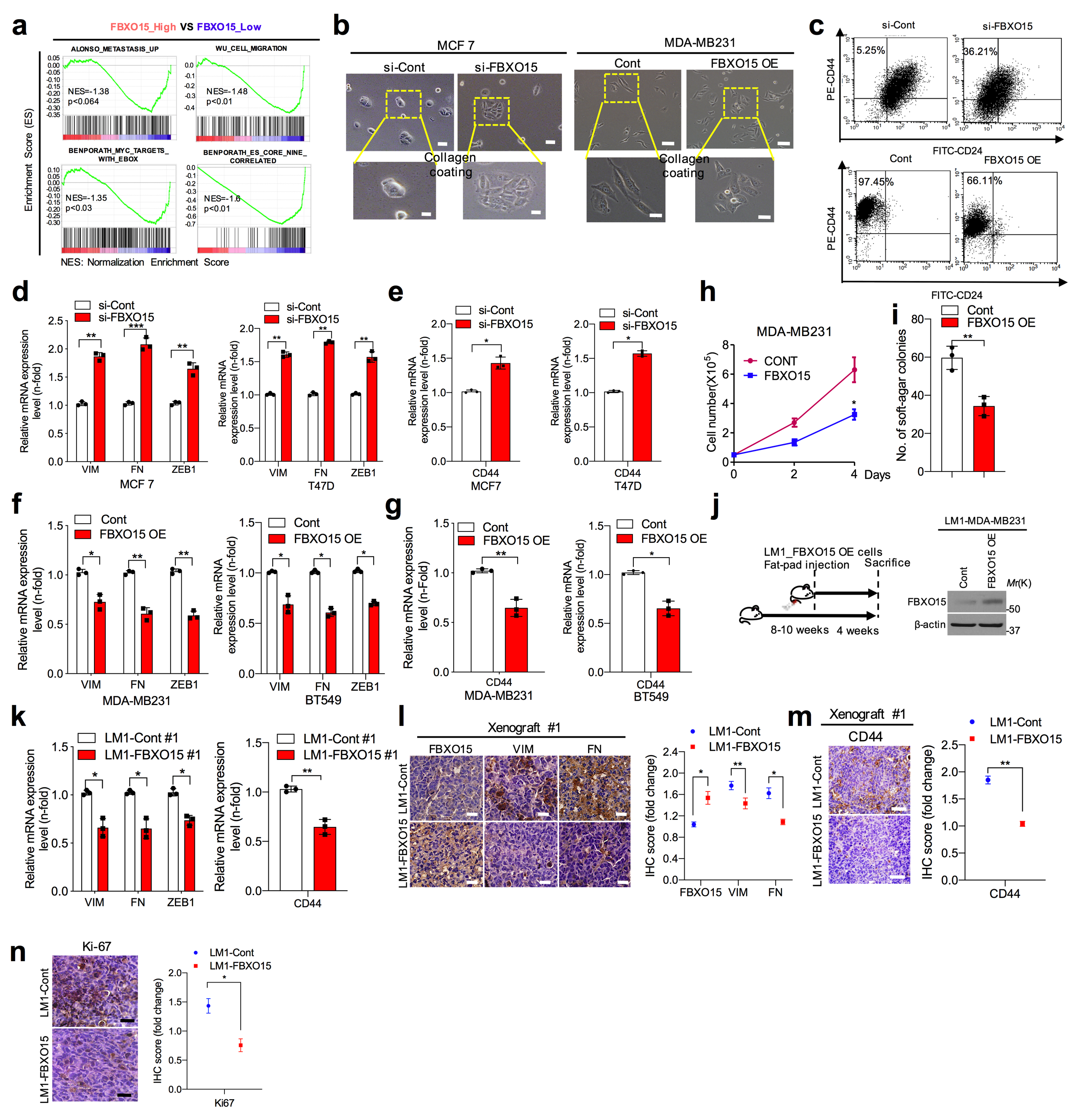


**Figure S2.** **FBXO15 regulates EMT and CSC both in vitro and in vivo. a.** Hallmarks of cancer progression analyzed using GSEA analysis, the data from GSE42568 database. GSEA analysis indicated that high expression of FBXO15 significantly downregulated genes involved in EMT and CSC. **b.** A collagen coating assay was performed to measure the cell morphology of the FBXO15-silenced MCF7 and FBXO15-overexpressing MDA-MB231 cells. Scale bar = 100 μm (upper), 50 μm (below). **c.** Flow cytometric analysis of the percentage of CD44+/CD24- cells in the FBXO15-silenced MCF7 and FBXO15-overexpressing MDA-MB231 cells. **d-g.** QRT-PCR analysis showing markers and regulators of EMT and CSC after knockdown or overexpressing of FBXO15 in MCF7 or T47D luminal type- and MDA-MB23 or BT549 basal type-breast cancer cells, respectively. **h, i.** Cell growth and soft agar assays were performed to show that overexpression of FBXO15 can inhibit MDA-MB-231 cell growth. **j.** Schematic experimental model for the mouse fat-pad injection of control and FBXO15-overexpressing lung-metastasized MDA-MB-231 stable cells (n=5) (left). The overexpression efficiency of FBXO15 was assessed by western blots (right). **k.** QRT-PCR analysis of EMT and CSC markers and regulators using mouse tissues. **l, m.** Representative images of IHC staining of FBXO15, VIM, FN and CD44 and the graph showing IHC staining scores. Scale bar = 100 μm. **n.** Representative images of IHC staining of Ki-67 in mouse tissue (left). Scale bar =100 µm. The graph shows the IHC staining score of Ki-67 (right). β-actin was used as a control for normalization of expression. *p < 0.05, **p < 0.001, and ***p < 0.0001; determined by two-tailed Student’s t-test (95% confidence).


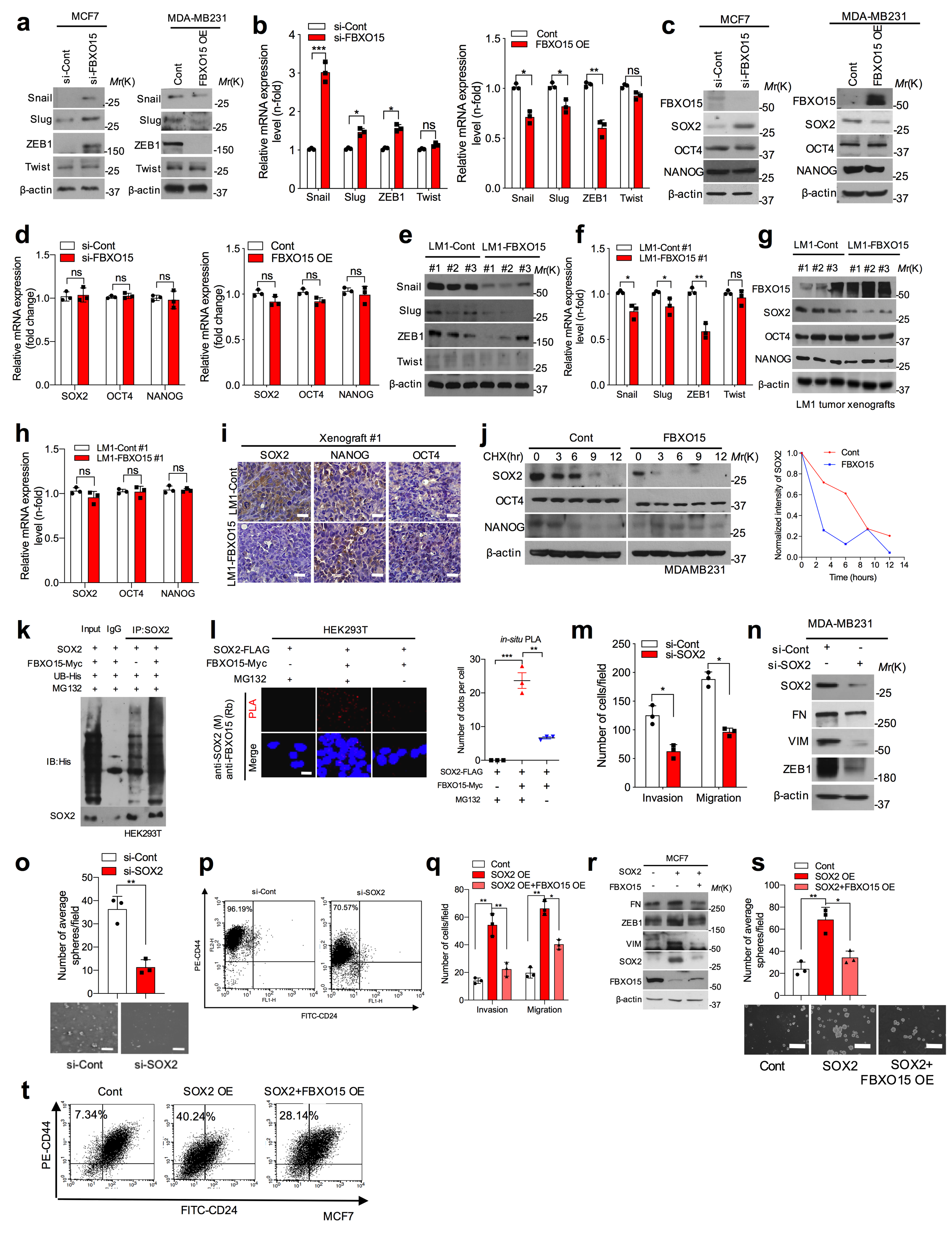


**Figure S3.** **SOX2 is a direct target of FBXO15 for proteasomal degradation.** **a, b.** Western blotting and qRT-PCR analysis of EMT regulators after knockdown or overexpression of FBXO15 in MCF7 and MDA-MB231 cells, respectively. **c.** Western blotting analysis of SOX2, OCT4, and NANOG protein expression in the FBXO15-silenced MCF7 or FBXO15-overexpressed MDA-MB231 cells. **d.** QR-PCR analysis of SOX2, OCT4, and NANOG mRNA expression after silencing or overexpressing FBXO15 expression in MCF7 and MDA-MB231 cells, respectively. **e, f.** Western blot and qRT-PCR analysis were performed using mouse tissues to check EMT regulators expression. **g, h.** western blotting and qRT-PCR analysis of SOX2, OCT4, and NANOG expression using mouse tissues. **i.** Representative images of IHC staining of SOX2, NANOG, and OCT4. Scale bar = 100 μm. **j.** CHX pulse chase assay showing the stability of SOX2, NANOG, and OCT4 after FBXO15 overexpression in MDA-MB231 cells (left). Representative graph of SOX2 expression quantification (right). **k.** In vivo ubiquitination assay of SOX2 using HEK293T cells in the presence or absence of FBXO15 expression, all of the samples were treated with MG132 (10 μM) for 6 hours before harvest. **l.** Representative confocal images and graph of cells with PLA-positive signal using HEK293T cells fixed with anti-SOX2 (M) and anti-FBXO15 (Rb). The signals in the image were counted and divided by the number of nuclei to obtain an average of signals per cell in each image using ImageJ software. **m.** Invasion and migration assays were performed to determine the cell numbers after SOX2 was blocked using siRNA in MDA-MB-231 cells. **n.** Western blotting analysis of EMT markers and regulators after SOX2 knockdown in MDA-MB-231 cells. **o, p.** Analysis of sphere formation and flow cytometry were performed using the cells transfected under the same conditions. **q.** The invasive and migrated cell numbers were assessed using SOX2-overexpressing MCF7 cells with or without FBXO15 expression. **r.** Western blotting analysis of EMT markers and regulators using the same rescue experiments condition. **s, t.** Sphere formation assays and flow cytometric analysis were performed after overexpressing SOX2 alone or together with FBXO15 in MCF7 cells. Scale bar = 100 μm. β-actin was used as a control for normalization of expression. *p < 0.05, **p < 0.001, and ***p < 0.0001; ns, not significant; determined by two-tailed Student’s t-test (95% confidence interval).


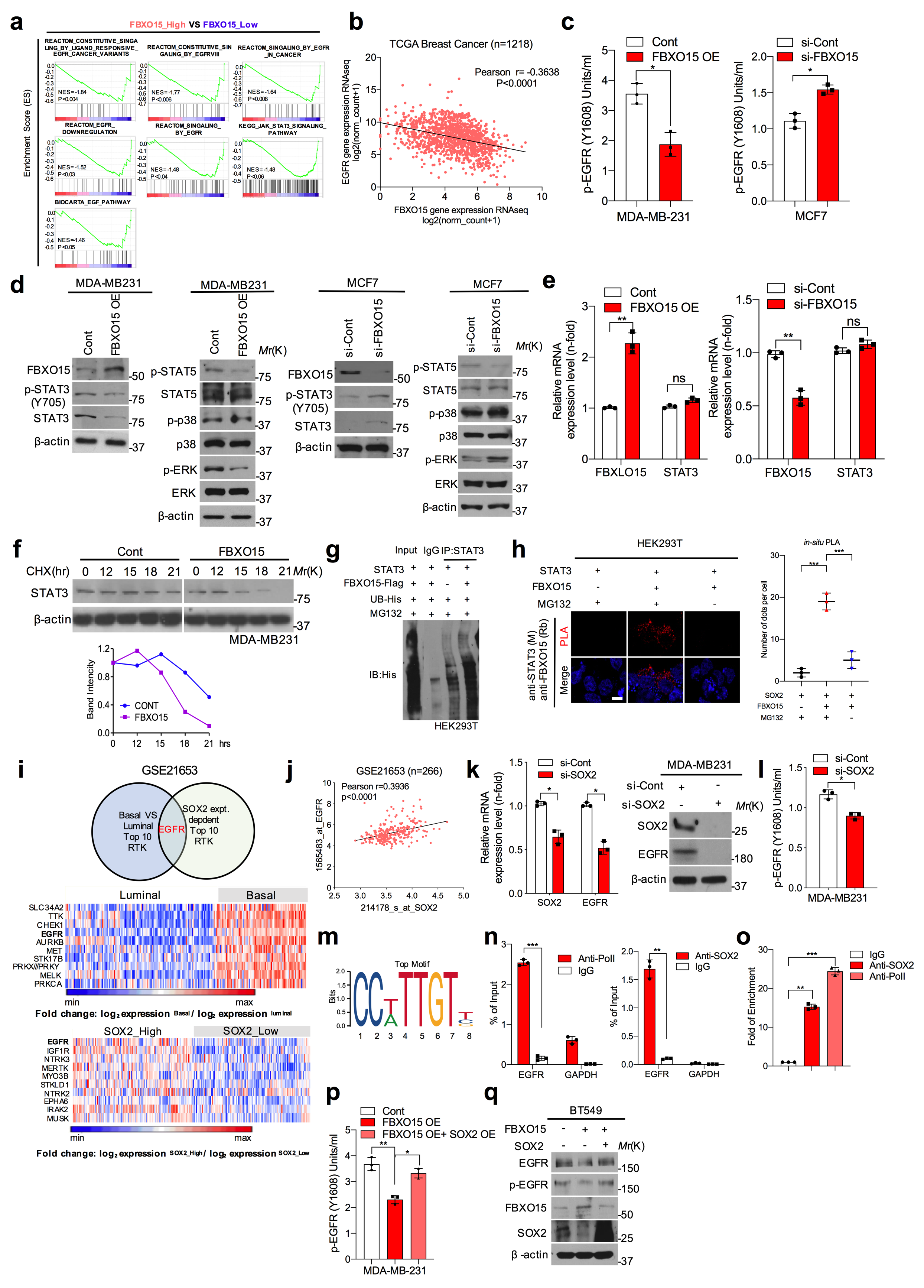


**Figure S4. FBXO15 downregulates EGFR expression and activation and directly interacts with STAT3 in breast cancer. a.** GSEA showed that high expression of FBXO15 negatively correlated with the EGFR-related signaling pathway among identified signaling pathways using a GSE42568 dataset from the GEO database (<https://www.ncbi.nlm.nih.gov/geo/)>. NES, normalized enrichment scores**. b.** A negative correlation between FBXO15 and EGFR was observed using the public TCGA database (n=1218). **c**. ELISAs analysis were performed to check p-EGFR expression levels after overexpressing FBXO15 in MDA-MB-231 cells or FBXO15-silenced MCF7 cells. **d,** Western blotting analysis were performed to check the main downstream effectors expression of EGFR after overexpressing or blocking FBXO15 in MDA-MB231 or MCF7 cells, respectively. **e.** qRT-PCR analysis were performed to assess the STAT3 mRNA expression level useing the same condition cell sets. **f.** CHX pulse chase assay showing the stability of STAT3 after FBXO15 overexpression in MDA-MB231 cells (upper). Representative graph of STAT3 expression quantification (below). **g.** In vivo ubiquitination assay of STAT3 using HEK293T cells in the presence or absence of FBXO15. All of samples were treated with MG132 (10 μM) for 6 hours before harvest. **h.** Representative confocal images and graph of cells with PLA-positive signal using HEK293T cells fixed with anti-STAT3 (M) and anti-FBXO15 (Rb). The signals in the image were counted and divided by the number of nuclei to obtain an average of signals per cell in each image using Image J software. Scale bar = 100 μm. **i.** Heatmaps showing the rank of RTKs expressing higher levels in basal-type than luminal-type breast cancers (upper) and the expression of RTKs which depend on SOX2 expression (below) using the GSE21653 breast cancer database. The overlap showed that EGFR was the only one that fitted these two criteria. Red and blue indicate upregulation and downregulation, respectively. **j.** A positive correlation was observed between SOX2 and EGFR mRNA expression using public data from the GSE21653 (n=266) database. **k.** qRT-PCR and western blotting analysis of SOX2 and EGFR expression at the mRNA and protein levels, respectively after SOX2 knockdown in MDA-MB-231 cells. **l.** ELISA was performed to assess EGFR activation after silencing SOX2 in MDA-MB-231 cells. **m.** The DNA binding consensus sequence of SOX2 from Chen et al. (2008a) and reanalyzed by JASPAR online tool (<http://jaspar.genereg.net)>. **n, o.** ChIP-qPCR analysis showed that SOX2 can directly bind to the specific site (CCTTTGTT) on the EGFR promoter. ChIP-qPCR data are presented as percentage of input and fold enrichment (see details in *Materials and Methods*). SD was calculated from qPCRs performed in triplicate. The primers were used as EGFR promoter Forward: 5’- GTTTCTACGGACTGCTCTCAGC- 3’, EGFR promoter Reverse: 5’- CCTGAACGGTGGGGTTTTTC- 3’; GAPDH Forward: 5’- TACTAGCGGTTTTACGGGCG- 3’. GAPDH Reverse: 5’- TCGAACAGGAGGAGCAGAGAGCGA- 3’. **p.** Rescue experiments were performed using ELISA assay to assess p-EGFR expression using FBXO15-overexpressing MDA-MB231 cells with or without SOX2 expression. **q.** Western blotting analysis using BT549, another basal type of breast cancer cell to check the EGFR and p-EGFR protein expression at the same rescue experiments condition. β-actin was used as a control for normalization of expres sion. *, p < 0.05; **, p<0.01; ***, p < 0.0001; ns, not significant.


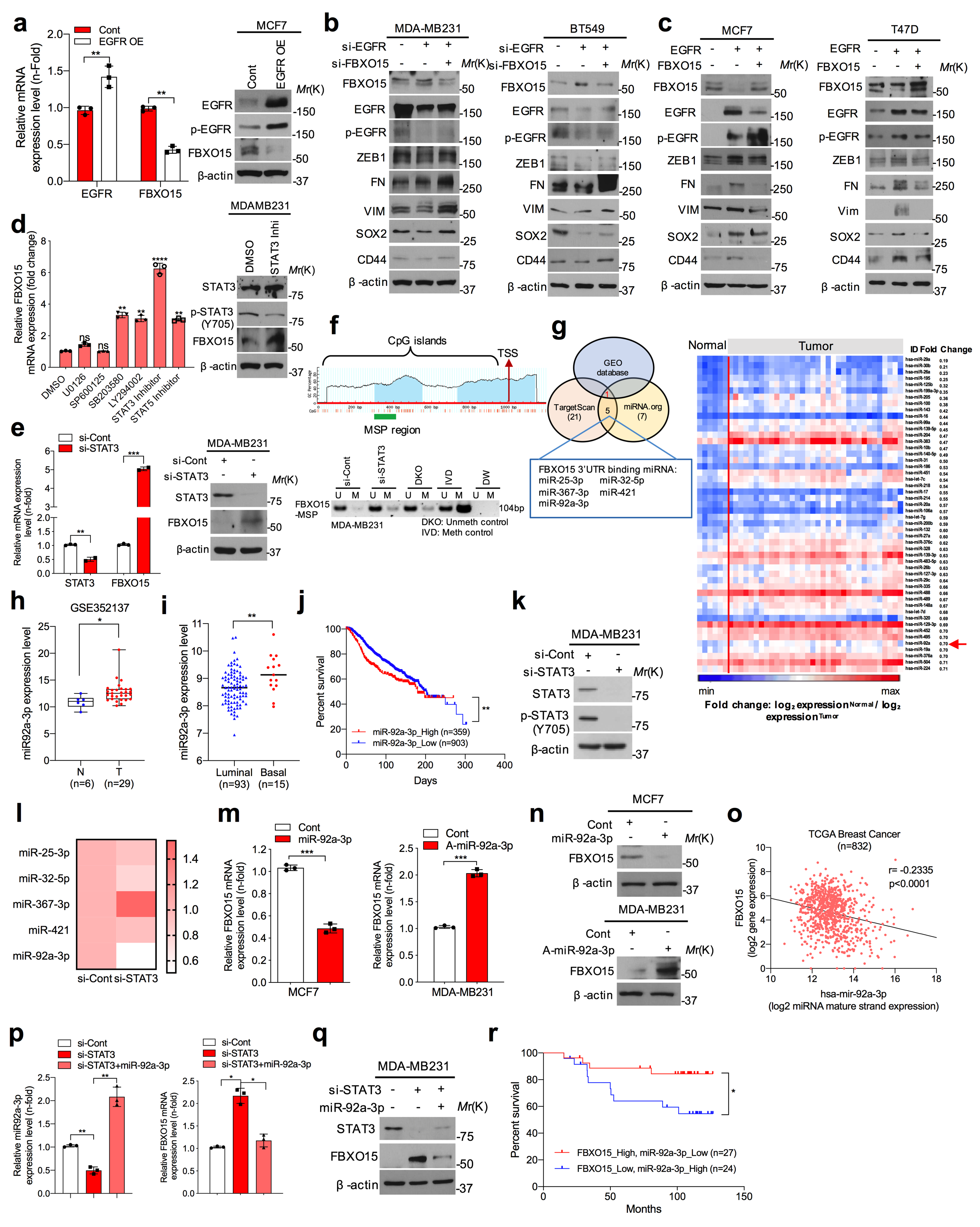


**Figure S5.** **The EGFR/STAT3/miR-92a-3p axis downregulates FBXO15 expression in breast cancer. a.** qRT-PCR and western blotting analysis of FBXO15 expression in EGFR-overexpressing MCF7 cells. **b, c.** Rescue experiments were performed using western blotting analysis with silencing EGFR alone or together with FBXO15 using si-RNA both in MDA-MB-231 and BT549 cells, respectively; or overexpressing EGFR with or without FBXO15 overexpression vector both in MCF7 and T47D cells, respectively. **d.** Detection of FBXO15 mRNA expression after treatment with U0126 (10 μM, ERK inhibitor), SP600125 (10 μM, MAPK inhibitor), SB203580 (25 μM, P38 inhibitor), LY294002 (10 μM, PI3K inhibitor), a STAT3 inhibitor (10 μM), and a STAT5 inhibitor (10 μM) in MDA-MB231 cells (left). Western blotting analysis of STAT3, p-STAT3, and FBXO15 expression after treatment with STAT3 inhibitor in MDA-MB231 cells (right). **e.** QRT-PCR and western blotting analysis of STAT3 and FBXO15 expression at mRNA (left) and protein levels (right) after transfecting si-RNA of STAT3 in MDA-MB231 cells. **f.** Schematic of the CpG island position in the FBXO15 promoter (upper). DNA methylation analysis in the CpG island of the FBXO15 promoter using bisulfite sequencing in genomic DNA isolated from MDA-MB231 cancer cells after transfection with si-control or si-STAT3 RNA (below). **g.** Schematic representation of miRNA prediction and screening using miRNA predictive websites (TargetScan and miRNA.org) and GSE35412 breast cancer dastabase (heatmap analysis, red and blue indicate upregulation and downregulation, respectively). **h.** GSEA (GSE35217) analysis showed that miR-92a-3p was upregulated in breast cancer patients (n=6) versus healthy individuals (n=29). **i.** A higher expression level of miR-92a-3p was observed in basal-type breast cancers than luminal-type breast cancers using data from GSE40525. **j.** Kaplan-Meier analysis of overall survival stratified by low and high miR-92a-3p expression in breast cancer patients. **k.** Efficiency analysis of STAT3 blocking in MDA-MB231 cells by western blotting analysis. **l.** QRT-PCR analysis of miR-25-3p, miR-32-5p, miR-367-3p, miR-421, and miR-92a-3p, expression after STAT3 knockdown in MDA-MB-231 cells showing by heatmap. **m, n.** QRT-PCR and western blotting analysis of FBXO15 expression after transfecting miR-92a-3p or miR-92a-3p inhibitor into MCF7 and MDA-MB231 cells, respectively. **o.** A negative correlation between miR-92a-3p and FBXO15 expression was observed using data from TCGA database (n=832). **p.** QRT-PCR analysis of miR-92a-3p and FBXO15 expression after transfecting si-STAT3 alone or together with miR-92a-3p into MDA-MB231 cells. **q.** Western blotting analysis of FBXO15 and STAT3 expression in treated MDAMB231 cells. **r.** Kaplan-Meier analysis showed that high expression levels of FBXO15 along with low levels of miR-92a-3p expression was associated with a longer survival rate of breast cancer patients. β-actin was used as a control for normalization of expression. *, p < 0.05; **, p < 0.01; ***, p < 0.001; ****, p < 0.0001; ns, not significant; determined by two-tailed Student’s t-test (95% confidence interval).

**References**

1 Rafferty, E. A. *et al.* Assessing radiologist performance using combined digital mammography and breast tomosynthesis compared with digital mammography alone: results of a multicenter, multireader trial. **266**, 104-113 (2013).

2 Katayama, K., Noguchi, K. & Sugimoto, Y. J. C. s. FBXO 15 regulates P‐glycoprotein/ABCB 1 expression through the ubiquitin–proteasome pathway in cancer cells. **104**, 694-702 (2013).

3 Liao, T. T. & Yang, M. H. J. M. o. Revisiting epithelial‐mesenchymal transition in cancer metastasis: the connection between epithelial plasticity and stemness. **11**, 792-804 (2017).

4 Mani, S. A. *et al.* The epithelial-mesenchymal transition generates cells with properties of stem cells. **133**, 704-715 (2008).

5 Kong, D. *et al.* Epithelial to mesenchymal transition is mechanistically linked with stem cell signatures in prostate cancer cells. **5**, e12445 (2010).

6 Wang, D. *et al.* Oct-4 and Nanog promote the epithelial-mesenchymal transition of breast cancer stem cells and are associated with poor prognosis in breast cancer patients. **5**, 10803 (2014).

7 Hadjimichael, C. *et al.* Common stemness regulators of embryonic and cancer stem cells. **7**, 1150 (2015).

8 Cui, C.-P. *et al.* Dynamic ubiquitylation of Sox2 regulates proteostasis and governs neural progenitor cell differentiation. **9**, 1-15 (2018).

9 Fang, L. *et al.* A methylation-phosphorylation switch determines Sox2 stability and function in ESC maintenance or differentiation. **55**, 537-551 (2014).

10 Zhao, L. *et al.* SGCE Promotes Breast Cancer Stem Cells by Stabilizing EGFR. 1903700.

11 Alanazi, I. O. & Khan, Z. J. A. P. J. C. P. Understanding EGFR signaling in breast cancer and breast cancer stem cells: overexpression and therapeutic implications. **17**, 445-453 (2016).

12 Dent, R. *et al.* Triple-negative breast cancer: clinical features and patterns of recurrence. **13**, 4429-4434 (2007).

13 Lee, C.-J. *et al.* Crosstalk between SOX2 and cytokine signaling in endometrial carcinoma. **8**, 1-12 (2018).

14 Johansson, H. & Simonsson, S. J. A. Core transcription factors, Oct4, Sox2 and Nanog, individually form complexes with nucleophosmin (Npm1) to control embryonic stem (ES) cell fate determination. **2**, 815 (2010).

15 Niwa, H., Ogawa, K., Shimosato, D. & Adachi, K. J. N. A parallel circuit of LIF signalling pathways maintains pluripotency of mouse ES cells. **460**, 118-122 (2009).

16 Loh, H.-Y. *et al.* The regulatory role of MicroRNAs in breast cancer. **20**, 4940 (2019).

17 Brock, M. *et al.* MicroRNA-18a enhances the interleukin-6-mediated production of the acute-phase proteins fibrinogen and haptoglobin in human hepatocytes. **286**, 40142-40150 (2011).

18 Liu, R., Zheng, H.-Q., Zhou, Z., Dong, J.-T. & Chen, C. J. J. o. B. C. KLF5 promotes breast cell survival partially through fibroblast growth factor-binding protein 1-pERK-mediated dual specificity MKP-1 protein phosphorylation and stabilization. **284**, 16791-16798 (2009).

19 Zhao, Y. *et al.* A Feedback Loop Comprising EGF/TGFα Sustains TFCP2-Mediated Breast Cancer Progression. **80**, 2217-2229 (2020).

20 Herman, J. G., Graff, J. R., Myöhänen, S., Nelkin, B. D. & Baylin, S. B. J. P. o. t. n. a. o. s. Methylation-specific PCR: a novel PCR assay for methylation status of CpG islands. **93**, 9821-9826 (1996).

21 Labidi-Galy, S. I. *et al.* Elafin drives poor outcome in high-grade serous ovarian cancers and basal-like breast tumors. **34**, 373-383 (2015).
